# Does Long-Term Use of Silver Nanoparticles Have Persistent Inhibitory Effect on *H. pylori* Based on Mongolian Gerbil's Model?

**DOI:** 10.1155/2014/461034

**Published:** 2014-04-17

**Authors:** Chao-Hung Kuo, Chien-Yu Lu, Yuan-Chieh Yang, Chieh Chin, Bi-Chuang Weng, Chung-Jung Liu, Yen-Hsu Chen, Lin-Li Chang, Fu-Chen Kuo, Deng-Chyang Wu, Hong-Lin Su

**Affiliations:** ^1^Division of Gastroenterology, Department of Internal Medicine, Kaohsiung Medical University Hospital, 100 Tz-You 1st Road, Kaohsiung City 807, Taiwan; ^2^Cancer Center, Kaohsiung Medical University Hospital, Kaohsiung City 807, Taiwan; ^3^Department of Medicine, Faculty of Medicine, College of Medicine, Kaohsiung Medical University, 100 Tz-You 1st Road, Kaohsiung City 807, Taiwan; ^4^Department of Laboratory Medicine, Kaohsiung Medical University Hospital, Kaohsiung City 807, Taiwan; ^5^Division of Infectious Diseases, Department of Internal Medicine, Kaohsiung Medical University Hospital, Kaohsiung City 807, Taiwan; ^6^Department of Microbiology, Kaohsiung Medical University, Kaohsiung City 807, Taiwan; ^7^School of Medicine, College of Medicine, E-Da Hospital, I-Shou University, Kaohsiung City 824, Taiwan; ^8^Division of Internal Medicine, Kaohsiung Municipal Hsiao-Kang Hospital, Kaohsiung City 812, Taiwan; ^9^Department of Life Sciences, National Chung Hsing University, 250, Kuo-Kuang Road, Taichung City 402, Taiwan

## Abstract

*Background*. It is urgent to find alternative agents due to increasing failure rate of *Helicobacter pylori (H. pylori*) eradication. The study surveyed the long-term effect of silver nanoparticles (AgNP) on *H. pylori* based on Mongolian gerbil's model. *Materials and Methods*. Fifty gerbils were randomly allocated to six groups (A–F). Group (Gr) A: the gerbils were fed with broth; Gr B and D: the gerbils were fed with AgNP/clay complex (0.1% of weight); Gr C and E: the gerbils were fed with AgNP/clay complex(1% of weight); and Gr D, E, and F: the gerbils were inoculated with *H. pylori*. At the 20th experimental week, the gerbils were sacrificed. Histology was evaluated according to the classification of the Sydney system. *P* < 0.05 was considered to be statistically significant. *Results*. The AgNP/clay has more obvious inhibitory effect on *H. pylori* in vitro. There was a trend of higher concentrations of AgNP with stronger inhibitory effect on *H. pylori* growth (*P* = 0.071). There were no significant differences of inflammation among groups D, E, and F (*P* = 0.688).*Conclusion*. AgNP/clay would be a potential and safe agent for inhibiting *H. pylori*. It should be helpful for eradication of *H. pylori* infection.

## 1. Introduction

*Helicobacter pylori (H. pylori) *infection is an important factor of many gastrointestinal diseases such as chronic gastritis, peptic ulcer, and gastric cancer. Eradication of* H. pylori* is the most important strategy for treatment of these diseases. However, the failure rate of currently used first-line therapies has increased up to 30% [1–4]. Therefore, there is an urgent need to find alternative agents to improve the efficacy.

The potential side effects of bismuth decrease the compliance of second-line eradication therapies. For the conventional antimicrobial treatments, some metals including silver in large quantities are applied to control skin infection [5], so previous studies applied silver to be the alternative treatment for gastrointestinal symptoms and infections (including* H. pylori*) [6]. Besides this, silver compounds have been used as antiulcer agents [7]. Because* H. pylori *is a major peptic-ulcer cause, it is logical that the antiulcer activity of silver might be related to its effects on* H. pylori*.

Currently, metal-based nanopreparations have become more and more important in their applications in various fields including antimicrobial abilities [8–14]. The synthesis of silver nanoparticles (AgNP) has aroused more interest because of the broad applications including wound dressings and medical devices [10, 11, 13, 15, 16]. The possible mechanism of action of metallic agents is the inhibition of* H. pylori *urease [17–19]. However, previous studies were mostly in vivo studies, so we need an ideal animal model to survey the long-term effects of AgNP exposure on human physiology and* H. pylori*. Watanabe et al. [20] demonstrated that* H. pylori* infection could induce well-differentiated adenocarcinoma based on a Mongolian gerbil's model. The Mongolian gerbils provide a suitable experimental animal model, so the aim of our study was to survey the long-term effect of AgNP on* H. pylori* based on a Mongolian gerbil's model.

## 2. Materials and Methods

Experiments were performed according to the experimental guidelines of the Ethics Committee of Kaohsiung Medical University Laboratory Animal Center.

### 2.1. Animals and Housing

Eight-week-old gerbils with body weight of 30–40 gm were purchased from the Kaohsiung Medical University Experimental Animals Center, Kaohsiung, Taiwan. In usual time, 4 to 5 gerbils per cage were housed and maintained under standard laboratory conditions (room temperature, 23°C~26°C; relative humidity, 55%~65%; 12/12-hour light/dark cycle) with free access to a commercial rodent diet and tap water.

### 2.2. Synthesis of AgNP/Clay Nanohybrid

The AgNP/clay complex was prepared via the reduction of silver ions in water according to the procedures reported previously [21]. In a typical experimental procedure, the lucentite SWN clay slurry (30 g, 1 wt% in water; DEUCHEM Co., Taiwan) was prepared by swelling in deionized water at 80°C and the AgNO_3_ solution (3.4 g, 1.0 wt% in water; J.T. Baker, USA) was then added to this slurry. The reducing agent was added to the AgNO_3_/clay solution and the mixture was vigorously stirred and heated at 80°C for 3 h. During the process, a color change was observed from yellowish to deep red, indicating the reduction of Ag^+^ to Ag^0^. The UV absorption at 420 cm^−1^ was observed using a UV-mini 1240 spectroscope. The Ag particle size on clay was measured with a field emission scanning electron microscope (FE-SEM, Zeiss EM 902A) at 80 kV. The d spacing was analyzed using a Shimadzu SD-D1 X-ray diffractometer with a Cu target (*γ* = 1.5405 Å) at a generator voltage of 35 kV, a generator current of 30 mA, and a scanning rate of 2°/min. The inorganic fraction was determined by decomposing the composites at temperatures up to 900°C. The concentration of dissolved Ag^+^ in solution was determined with inductively coupled plasma mass spectrometry (ICP-MS) provided in National Sun Yat-Sen University and National Tsing Hua University of Taiwan. The supernatant of a 0.1 wt% AgNP/clay sample in solution was collected after centrifugation at 16,000 ×g for 30 min. ICP-MS analysis showed the Ag^+^ concentration to be in a range of 139.33 ± 16.04 ppb. After adding 3% HNO_3_ to the supernatant to convert the free Ag^0^ to Ag^+^, the concentration increased to 155.33 ± 34.53 ppb. [15]

### 2.3. Dose Escalation of AgNP

Part I: the optimal concentration of the antibacterial activity of AgNP/clay: AgNP/clay (0.06%, 0.08%, 0.1%, 0.2%, and 0.3% of weight) was added into the Brucella broth containing bacteria 1 × 10^3^ CFU/mL and then they were incubated at 37°C. The incubation time was 12 hours. Then 100 *μ*L of these cultured broths were spread on CDC Anaerobe 5% Sheep Blood Agar (BD, USA) and incubated at 37°C with 5% O_2_ conditions. The colony numbers were counted after 48 hours of incubation. Part II: the time course of the antibacterial activity of AgNP/clay: AgNP/clay (0.01%, 0.05%, and 0.1% of weight) was added into the Brucella broth containing bacteria 1 × 10^3^ CFU/mL and then they were incubated at 37°C. The incubation time was 0, 0.5, 1, 2, 4, 12, and 24 hours, irrespectively. Then 100 *μ*L of these cultured broths was spread on CDC Anaerobe 5% Sheep Blood Agar (BD, USA) and incubated at 37°C with 5% O_2_ conditions. The colony numbers were counted after 48 hours of incubation.

### 2.4. *H. pylori* Inoculation

**Figure 1:**
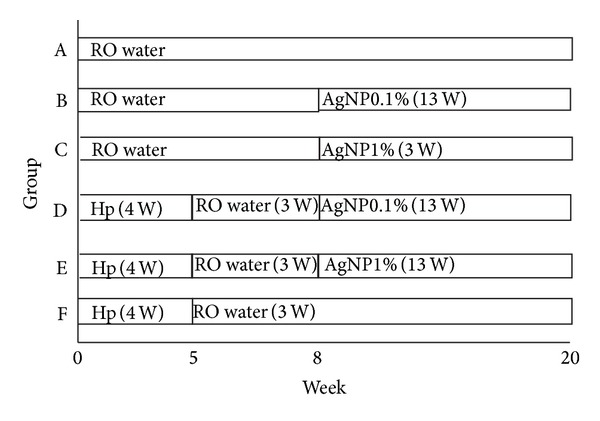
The timing of AgNP/clay and* H. pylori* given. Group (Gr) A: the gerbils were fed with broth only. Gr B and D: the gerbils were fed with AgNP/clay (0.1% of weight) in the 8th to 20th week. Gr C and E: the gerbils were fed with AgNP/clay (1% of weight) in the 8th to 20th week. Gr D, E, and F: the gerbils were inoculated with* H. pylori *[CagA (+)/VacA (+)] in the 1st to 4th week. At the end of the 20th experimental week, the animals were fasted for 24 hours before being sacrificed.

The gerbils were randomly allocated to six groups according to a randomized number (A–F): group A: the gerbils were fed with broth only; group B: the gerbils were fed with AgNP/clay complex (0.1% of weight) in the 8th to 20th week; group C: the gerbils were fed with AgNP/clay complex (1% of weight) in the 8th to 20th week; group D: the gerbils were inoculated with* H. pylori *[CagA (+)/VacA (+)] during the 1st to 4th week and then they were fed with AgNP/clay complex (0.1% of weight) in the 8th to 20th week; group E: the gerbils were inoculated with* H. pylori *[CagA (+)/VacA (+)] during the 1st to 4th week and then they were fed with AgNP/clay complex (1% of weight) in the 8th to 20th week; group F: the gerbils were inoculated with* H. pylori *[CagA (+)/VacA (+)] during the 1st to 4th week. At the end of the 20th experimental week, the animals were fasted for 24 hours before being sacrificed (Figure 1).

### 2.5. Histological Evaluation of the Gastric Mucosa in Gerbils

Samples of the gastric mucosa were excised from each gerbil stomach for the assessment of the presence of* H. pylori *and gastric inflammation using Giemsa and hematoxylin-eosin (HE) staining for histological examination, respectively. The samples were fixed in 10% buffered formalin and embedded in paraffin [II-34]. The paraffin sections were cut at a thickness of 5 mm and stained. Two experienced pathologists, blinded to the treatment given, performed histological examinations. Histological features of mucosal inflammation and intestinal metaplasia were evaluated for each specimen under a light microscope according to the classification of the Sydney system. The degree of inflammatory cell infiltration and the area of intestinal metaplasia were scored as follows: 0, normal; 1, mild; 2, moderate; 3, marked.

### 2.6. Statistical Analyses

We analyzed the collected data using the statistical software package SPSS. Kruskal-Wallis test was used for comparing histological change of mucosa. *P* < 0.05 was considered to be statistically significant.

## 3. Results

**Figure 2:**
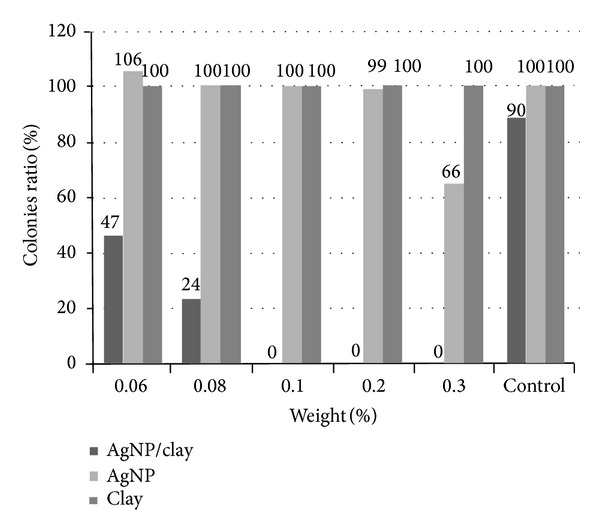
The inhibitory effects of different materials on* H. pylori *were surveyed. The results revealed that AgNP/clay has more obvious inhibitory effect on* H. pylori *(concentration more than 0.08% of weight). However, clay did not have the inhibitory effect and the AgNP had mild inhibitory effect only in high concentrations.* H. pylori: Helicobacter pylori;* AgNP: silver nanoparticles.

The inhibitory effects of different materials on* H. pylori *were surveyed. The results revealed that AgNP/clay has more obvious inhibitory effect on* H. pylori*. This inhibitory effect became more obvious when the concentration of AgNP/clay was more than 0.08% of weight. However, the clay did not show any inhibitory effect in any concentration. The AgNP had mild inhibitory effect only at high concentration (0.3% of weight) (Figure 2).

**Figure 3:**
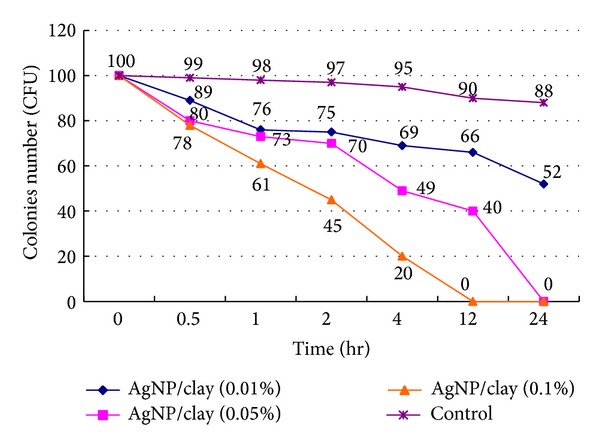
We surveyed the reaction time of inhibiting* H. pylori* in different concentrations of AgNP/clay. We found that the higher the concentration, the shorter the reaction time.* H. pylori *could be completely inhibited within 12 hours in a concentration of 0.1% of weight.* H. pylori: Helicobacter pylori;* AgNP: silver nanoparticles.

We surveyed the reaction time of inhibiting* H. pylori* in different concentrations of AgNP/clay. We found that the higher the concentration of AgNP/clay, the shorter the reaction time. In concentration of 0.1% weight of AgNP/clay,* H. pylori* would be completely inhibited since the 12th hour and this effect would persist up to the 24th hour (Figure 3). So the optimal frequency of feeding AgNP/clay might be daily.

Fifty gerbils were used in this study. The numbers of gerbils in each group were 6 (Gr A), 6 (Gr B), 6 (Gr C), 11 (Gr D), 15 (Gr E), and 6 (Gr F), respectively. The study design is shown in Figure 1. In our study, all gerbils were alive till the end of this experiment, and there was no significant difference in the survival rates among the various groups. On the 13th week, the positive rates of* H. pylori* were all 100% in groups D, E, and F.

**Figure 4:**
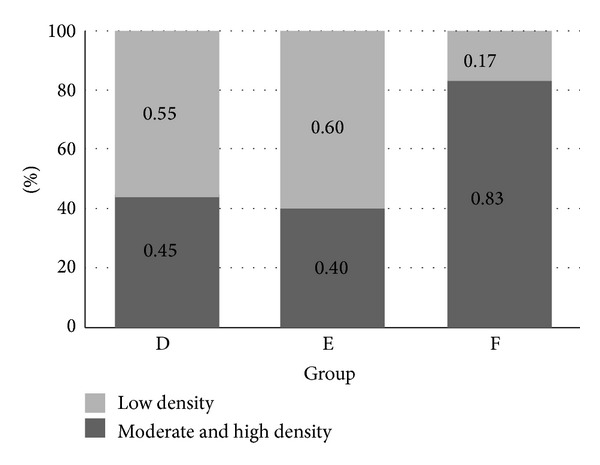
The status of* H. pylori* densities in groups D, E, and F is shown. Group F had obviously higher proportion of moderate and high* H. pylori* densities. It disclosed that AgNP/clay had an inhibitory effect on* H. pylori*.* H. pylori: Helicobacter pylori;* AgNP: silver nanoparticles.

We surveyed the densities of* H. pylori* in groups D, E, and F. We wanted to survey the inhibitory effect of AgNP on* H. pylori*. The average densities of* H. pylori* (according to Sydney classification) were 1.45 ± 0.52, 1.27 ± 0.70, and 1.83 ± 0.41 in Gr D, E, and F, respectively. It did not show significant difference between the three groups (*P* = 0.071); however, we found the trend that higher concentrations of AgNP had stronger effect on inhibiting* H. pylori* (Figures 4 and 6).

**Figure 5:**
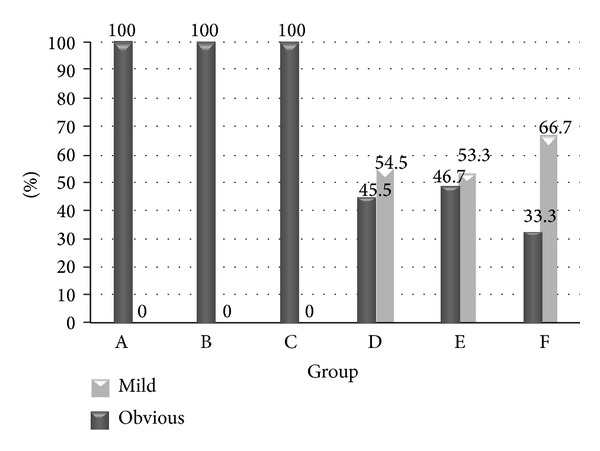
The inflammatory severities of gerbils' stomach in different groups are shown. All gerbils in groups A, B, and C showed mild inflammation only. The proportion of obvious inflammation was more than mild inflammation in groups D, E, and F. The difference was the most obvious in group F. However, there was no significant difference among these groups.

**Figure 6:**
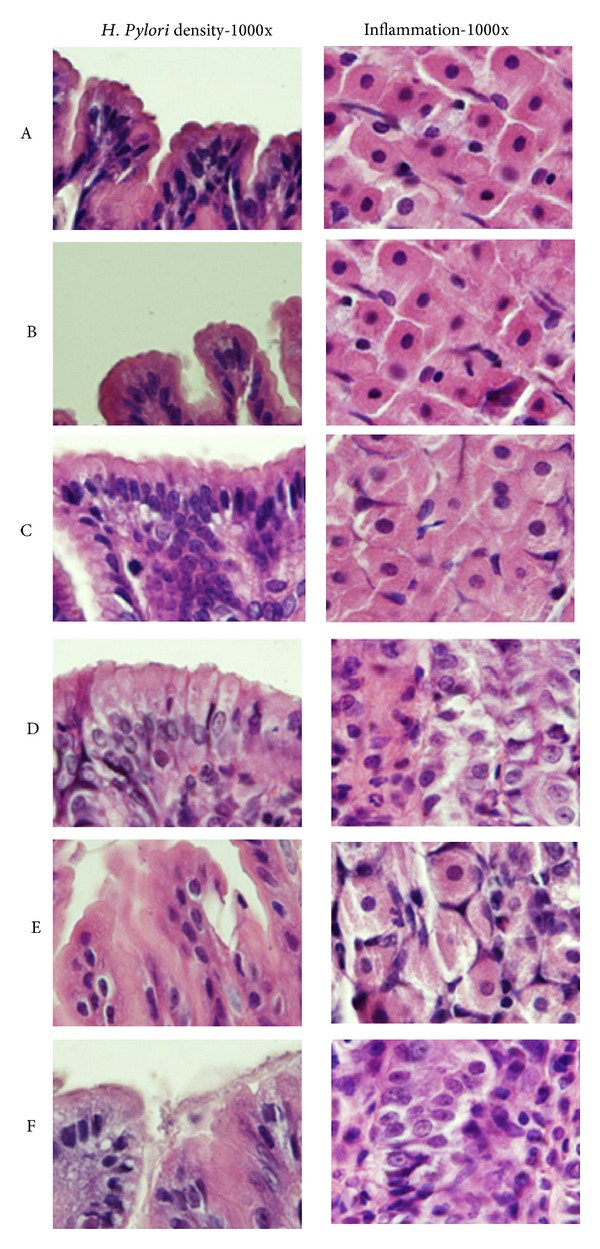
Histological changes of different groups are shown (H&E stain 1000x). We show the status of* H. pylori* density in right field and the status of inflammation in left field. There is no obvious inflammatory cells infiltration in groups A, B, and C, but obvious inflammatory cells infiltration was noted in groups D, E, and F. The densities of* H. pylori* were higher in group F.

The possible toxic effect of AgNP on gastric mucosa and its interaction with* H. pylori* were also surveyed. We showed the severity of inflammation of gerbil's mucosa according to the Sydney classification. There was no sign of inflammation noted in group A. However, all gerbils in groups B and C had mild monocyte infiltration but without neutrophil infiltration. The results of the inflammatory scores were 0 ± 0 (Gr A), 1.00 ± 0 (Gr B), 1.00 ± 0 (Gr C), 4.27 ± 1.10 (Gr D), 4.20 ± 1.82 (Gr E), and 4.83 ± 1.17 (Gr F). The proportions of moderate to severe inflammation were 0% (0/6), 0% (0/6), 0% (0/6), 54.5% (6/11), 53.3% (8/15), and 66.7% (4/6) in groups A, B, C, D, E, and F (Figures 5 and 6). The results revealed that AgNP alone did not have an acute toxic effect on gerbil's gastric mucosa. Besides these, there were no significant differences of inflammation among groups D, E, and F (*P* = 0.688).

## 4. Discussion

The approaches to overcoming drug resistance include increasing the dosage and treatment duration of drugs and using multiple drugs or pretreatment with agents to reduce bacterial load. The agents used to decrease* H. pylori* load include probiotics, bismuth, or some herbs. Our study was designed to survey whether AgNP could reduce the* H. pylori* load in gerbils.

To the best of our understanding, the present investigation can be considered to be the first report of the anti-*H. pylori *activity of silver nanoparticles based on a gerbil model. A previous study showed that silver nanoparticles may have the effect of inhibiting urease activity of* H. pylori *[19]. However, these previous studies were under the evidence of in vitro studies. We chose 13 weeks as the intervention's duration according to our previous preliminary data (not published) of Mongolian gerbil's model. We found that* H. pylori* would induce obvious inflammation since this time point. So we expected that there might be obvious difference in different groups at this time point.

According to our results, AgNP could not completely inhibit the growth of* H. pylori* neither in low nor high concentrations of AgNP. But AgNP showed the potential effect of decreasing densities of* H. pylori* and also had dose-dependent response. This supported the notion that AgNP might be a bacteriostatic agent for* H. pylori*. Our results supported the finding of a previous study [19]. The effect of inhibition was directly related to AgNP/clay itself but not free silver ion in solution, because the synthesized AgNP/clay was nearly free of Ag^+^ leaching from the nanohybrid, even after a storage period of six months at room temperature.

Multiple investigations have been performed to show the antibacterial activity of metals and metals chelated with some ligands against* H. pylori* [22, 23]. According to a previous report, the charged clay could trap bacteria and the close interfacial interaction between bacteria and AgNP plays a cardinal role in inhibiting bacteria [15]. The contact and interaction between the AgNP/clay and the cell wall of bacterium is important to trigger a cytotoxic signal; unfortunately,* H. pylori* was colonized within the gel layer of gastric mucosa and we thought that the gel layer might interfere with the contact between* H. pylori* and AgNP/clay. This should be one of the reasons in which* H. pylori* was not inhibited obviously in our gerbil's model.

A previous study found that under anaerobic conditions, newly synthesized AgNP/clay still enabled suppression of cell growth as efficiently as under aerobic conditions [15]. So its effect would not be influenced by the microanaerobic environment in which* H. pylori* lives. Another possible challenge is the acidic environment in the stomach; one study revealed that the bactericidal effect would diminish in an acidic environment [24]. This might be one of the reasons that AgNP/clay only had bacteriostatic effect in the gerbil's stomach.

The bacterial load is an important factor for eradication of* H. pylori*. Previous studies demonstrated that decreasing the amount of* H. pylori* would increase the success rate of eradication [25]. They usually used bismuth as the agent for inhibiting* H. pylori*, but the side effects of bismuth were obvious and would decrease the compliance and success rates. Another method might use more complex regimens, such as sequential therapies, to achieve successful eradication by lower bacterial load. In our study, we found that AgNP had the effect of inhibiting* H. pylori.* Our study might provide another reliable way to help us eradicate* H. pylori.*

A previous study in vitro demonstrated that urease inhibitory activity increased linearly with increased concentration of AgNP. So we tried two different doses of AgNP/clay (1% weight versus 0.1% weight) to survey the dose-dependent response. On the other hand, the possible toxic effect of AgNP was an important concern of this study. Many studies have revealed AgNP to have mild toxicity against several cell lines and the possible mechanism of these toxic effects is under survey [26–28], so our study also surveyed the toxic effect on gastric mucosa. In our results, no obvious mucosal damage was found in groups B and C. According to previous study [6], the paper clearly demonstrated that fed silver nanoparticles were not absorbed by animal. In our study, we did not find any evidence about AgNP absorbed by gastrointestinal mucosa. However, further survey and longer observation period would be needed for application of AgNP in human.

There were some limitations of our study. One was the duration of feeding AgNP. To our best knowledge, there is no previous similar design reported in Mongolian gerbil's model, so we did not know the optimal intervention period. It might need longer therapeutic period for obtaining obvious inhibitory effect. Another limitation was the unequal number of gerbils used in these groups. However, we used more gerbils in groups D and E (fed both* H. pylori* and AgNP). The number per group of gerbils used in previous studies was around six to ten, so the number of gerbils used in our study groups A, B, C, and F was enough for analysis. Besides this, we kept feeding gerbils during the experiment and the various ions in the food might have interaction with AgNP/clay. So the effect of AgNP/clay on* H. pylori* was possibly diminished.

In summary, our study showed that AgNP/clay would be a potential and safe agent for decreasing the amount of* H. pylori*. It should be helpful for eradication of* H. pylori* infection and needs further survey on the method given.
